# Correction with blood T1 is essential when measuring post-contrast myocardial T1 value in patients with acute myocardial infarction

**DOI:** 10.1186/1532-429X-15-11

**Published:** 2013-01-19

**Authors:** Eui-Young Choi, Sung Ho Hwang, Young Won Yoon, Chul Hwan Park, Mun Young Paek, Andreas Greiser, Hyemoon Chung, Ji-Hyun Yoon, Jong-Youn Kim, Pil-Ki Min, Byoung Kwon Lee, Bum-Kee Hong, Se-Joong Rim, Hyuck Moon Kwon, Tae Hoon Kim

**Affiliations:** 1 Division of Cardiology, Heart Center, Gangnam Severance Hospital Yonsei University College of Medicine, Seoul, South Korea; 2 Department of Radiology Gangnam Severance Hospital, Yonsei University College of Medicine, Seoul, South Korea; 3 Siemens Healthcare, Seoul, Republic of Korea; 4 Siemens AG Healthcare, Erlangen, Germany

**Keywords:** Cardiovascular magnetic resonance, T1 mapping, Myocardium

## Abstract

**Background:**

Post-contrast T1 mapping by modified Look-Locker inversion recovery (MOLLI) sequence has been introduced as a promising means to assess an expansion of the extra-cellular space. However, T1 value in the myocardium can be affected by scanning time after bolus contrast injection. In this study, we investigated the changes of the T1 values according to multiple slicing over scanning time at 15 minutes after contrast injection and usefulness of blood T1 correction.

**Methods:**

Eighteen reperfused acute myocardial infarction (AMI) patients, 13 cardiomyopathy patients and 8 healthy volunteers underwent cardiovascular magnetic resonance with 15 minute-post contrast MOLLI to generate T1 maps. In 10 cardiomyopathy cases, pre- and post-contrast MOLLI techniques were performed to generate extracellular volume fraction (Ve). Six slices of T1 maps according to the left ventricular (LV) short axis, from apex to base, were consecutively obtained. Each T1 value was measured in the whole myocardium, infarcted myocardium, non-infarcted myocardium and LV blood cavity.

**Results:**

The mean T1 value of infarcted myocardium was significantly lower than that of non-infarcted myocardium (425.4±68.1 ms vs. 540.5±88.0 ms, respectively, p< 0.001). T1 values of non-infarcted myocardium increased significantly from apex to base (from 523.1±99.5 ms to 561.1±81.1 ms, p=0.001), and were accompanied by a similar increase in blood T1 value in LV cavity (from 442.1±120.7 ms to 456.8±97.5 ms, p<0.001) over time. This phenomenon was applied to both left anterior descending (LAD) territory (from 545.1±74.5 ms to 575.7±84.0 ms, p<0.001) and non-LAD territory AMI cases (from 501.2±124.5 ms to 549.5±81.3 ms, p<0.001). It was similarly applied to cardiomyopathy patients and healthy volunteers. After the myocardial T1 values, however, were adjusted by the blood T1 values, they were consistent throughout the slices from apex to base (from 1.17±0.18 to 1.25±0.13, p>0.05). The Ve did not show significant differences from apical to basal slices.

**Conclusion:**

Post-contrast myocardial T1 corrected by blood T1 or Ve, provide more stable measurement of degree of fibrosis in non-infarcted myocardium in short- axis multiple slicing.

## Background

Post-contrast T1 mapping using modified Look-Locker inversion recovery sequence has been introduced as a promising means to assess expansion of the myocardial extra-cellular space even in the absence of scarring [1]. It can be used in various diseases such as acute or chronic myocardial infarction, various cardiomyopathies, valvular heart disease, or any disease that can cause myocardial fibrosis [2,3]. As the degree of left ventricular (LV) myocardial fibrosis can differ from segment to segment, especially in patients with ischemic heart disease, multiple slicing is needed to see regional differences in T1 values. However, post-contrast T1 value can change over time after bolus contrast injection [4,5]. To date, many studies have used T1 values measured 15 minutes after bolus contrast injection, assuming that this is the best time during which little change in T1 over time is observed [4,5]. But it has not been reported whether T1 value changes over time during acquisition of multiple short-axis images from apex to base. This question requires investigation because T1 mapping provides a potentially sensitive index for characterizing non-enhanced myocardium in cardiomyopathy, peri-infarction zones, or remote non-infarcted myocardium. Therefore, we investigated whether T1 values at 15 minutes after bolus contrast injection are stable during the period of short-axis slicing from apex to base. We also evaluated correction methods to provide better time-independent results during multiple short-axis slicing.

## Methods

Eighteen reperfused acute myocardial infarction (AMI) patients underwent cardiovascular magnetic resonance (CMR) including 15 minutes post-contrast MOLLI sequence to generate T1 maps. T1 maps consisted of six consecutive slices of the LV short axis from apex to base. Regions of interest (ROIs) were placed on whole myocardium, infarcted myocardium, non-infarcted myocardium, and the LV blood cavity on each slice. Then T1 values were generated using fitting curves. As positive controls, 13 cardiomyopathy (hypertrophic or dilated) patients underwent CMR with the same 15 minutes post-contrast MOLLI scanning and 10 of them underwent both pre- and post-15-minutes post contrast MOLLI. Blood hematocrit level were measured and used for calculating of extracellular volume fraction (Ve). As controls, eight healthy volunteers (without cardiovascular disease) underwent CMR with identical pre- and post-contrast MOLLI sequence. Four AMI patients underwent repeated scanning with 15 minute post-MOLLI in a different date. This study protocol was approved by our Institutional Review Board.

### MR imaging protocols

CMR was performed with a 1.5-T MR scanner (MAGNETOM Avanto; Siemens Medical Solutions, Erlangen, Germany) with an anterior 12-element phased array cardiac coil without posterior element, a maximum strength of 45 mT/m gradient system, and a maximum slew rate of 200 mT/m/s. Localization of the heart was performed with true fast imaging with steady-state precession (FISP) under electrocardiographic gating. Global ventricular function was evaluated using a FISP sequence along the short axis of the heart. The acquisition parameters were: repetition time (TR) = 55 ms, echo time (TE) = 1.1 ms, flip angle = 67°, 25 phases, slice thickness = 8 mm, slice gap = 2 mm, acquisition matrix = 192 × 109, and field of view 320 × 400 mm.

A prototype MOLLI sequence was applied before contrast injection with six short-axis slices to encompass the enhanced myocardium visualized on the late gadolinium enhancement (LGE)-CMR during breath-hold at full expiration in cases with cardiomyopathy. The injured myocardium was evaluated using LGE-CMR with a phase-sensitive inversion recovery prepared fast gradient echo (FGRE) sequence in 10 minutes after administration of 0.1 mmol/kg of a gadolinium-based contrast agent (gadoterate dimeglumine; Dotarem, Guerbet, France). The LGE-CMR was obtained in the same axis and slice thickness as for cine images. A contrast media bolus was intravenously administered at 2 mL/s, followed by 20 mL of normal saline at 4 mL/s, through a 20-gauge cannula in the antecubital vein using a power injector (Nemoto; Nemoto Kyorindo, Tokyo, Japan). The appropriate inversion time before LGE-CMR was determined using a FGRE sequence with inversion times varying from 150 to 650 msto nullify the signal from the normal myocardium. The LGE-CMR parameters were: TR = 600 ms, TE = 3.4 ms, flip angle = 25°, acquisition matrix = 256 × 156; and field of view 320 × 400 mm.

After LGE imagimg, a MOLLI sequence for post-enhancement myocardial T1 mapping was applied 15 minutes after gadolinium injection with slice position to LGE imaging. The MOLLI pulse sequence was a single-slice, single-shot, true fast image with a FISP technique that produced three, three, and five single shot readouts using three different inversion time blocks incremented by subsequent heart beats with a non-slice-selective 180° pulse, according to the heart rate. In each of the three Look-Locker (LL) experiments, the first single-shot readout was performed at TI (LL1 = 100 ms, LL2 = 180 ms, and LL3 = 260 ms) after a non-slice-selective adiabatic 180° pulse and at trigger delay time after the previous R-wave. Subsequent images were acquired at time trigger delay after every R-wave, until the final number of images for each LL experiment was acquired. [4] To reduce motion artifacts, a motion correction algorithm, provided by the vendor was applied. The key idea is estimating the synthetic images presenting similar contrast changes to original images by solving an energy minimization problem [6]. The imaging parameters were: slice thickness = 8 mm, TR = 740 ms, TE = 1.06 ms, flip angle = 35°, acquisition matrix = 192 × 124 and field of view = 320 × 400 mm. Then vendor generated T1 map by combining different 11 TI images (Figure 1).

**Figure 1:**
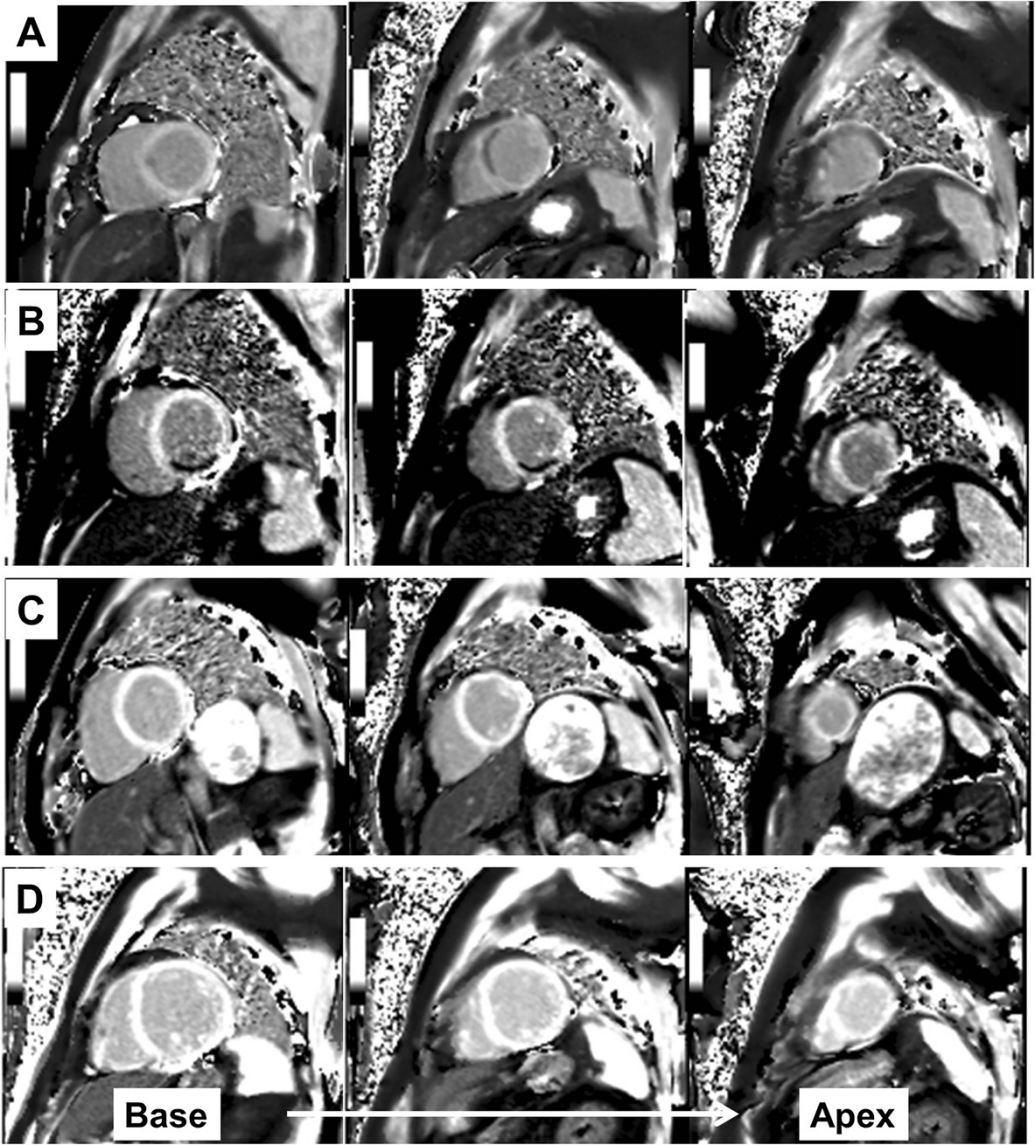
**Short**-**axis slices of reconstructed T1 maps from patients with left anterior descending coronary artery territory acute myocardial infarction (A), right coronary artery territory infarction (B), from a healthy control individual (C), and a patient with dilated cardiomyopathy (D).**

### Measurement of pre- and post-contrast T1 and Ve

ROIs were located on in one TI image which provides best contrast then the software (Qmass MR 7.5, Medis, Leiden, The Netherlands) automatically copied the ROI. From the 11 images with different inversion times, a T1 time-fitting curve was drawn with signal intensity in the vertical axis and inversion time in the horizontal axis, T1 relaxation time was generated based on a T1 time-fitting curve. T1 time-fitting algorithm was as follows; y=A -B exp (- t/*T*1*) where t is accumulative time from inversion and T1* is modified T1 in an LL experiment. Then T1 was calculated from the equation, T1=T1*[(B/A)-1] [1,4,7,8]. The ROIs were placed on whole myocardium, infarcted myocardium, non-infarcted myocardium, and LV blood cavity in AMI cases. In cardiomyopathy cases and healthy controls, ROIs were placed on whole myocardium and LV blood cavity (Figure 2). From each slice, corrected myocardial T1 value, defined as myocardial T1 value divided by corresponding LV cavity blood T1 value, was also measured. In healthy controls, sixteen segmental analyses [9] of post-contrast T1 value were also done. Ve was calculated in cardiomyopathy patients using the equation of; Ve (%)= ρ(100-hematocrit)×(1/T1_myo post_-1/T1_myo pre_)/(1/T1_blood post_-1/T1_blood pre_)–Vp; where ρ=specific density of myocardial tissue (1.05 g/ml) and Vp=myocardial plasma volume fraction (assuming 4.5% reflecting capillary density) [10,11].

**Figure 2:**
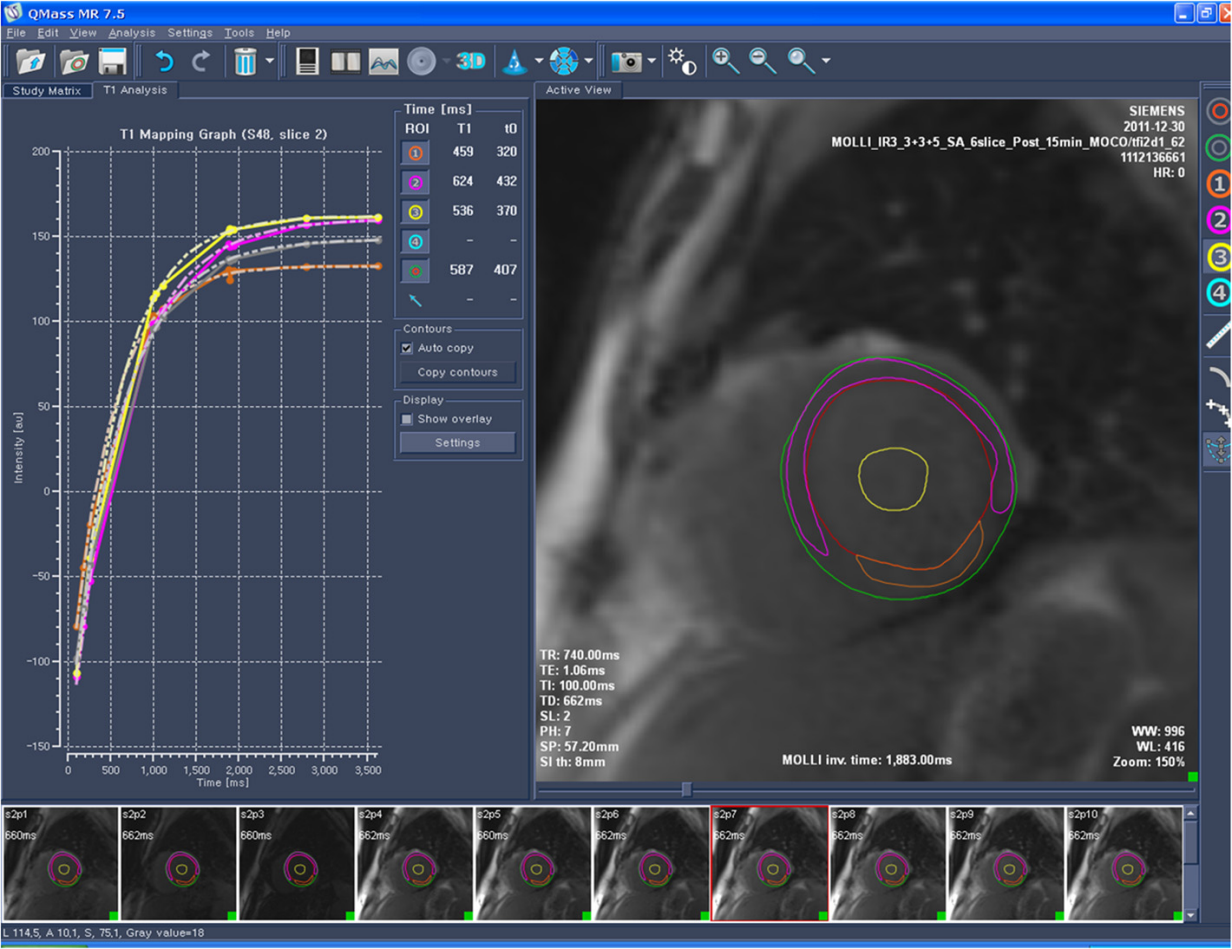
Measurement of T1-relaxational time from whole-, infarcted-, and non-infarcted myocardium, and left ventricular cavity blood from a T1 map.

### Statistical analysis

Continuous variables are presented as mean ± standard deviation and categorical variables are presented as number or percentages. Change of myocardial T1 value, myocardial/blood T1 ratio, and Ve according to slicing level from apex to base were analyzed by repeated measure ANOVA. Correlation analysis was done between age and T1 value of non-infarcted mid-LV slice in AMI patients and healthy controls. Difference of post-contrast T1 values between repeated scanning was analyzed by paired *t*-test. Difference of segmental T1 in a slice was analyzed by ANOVA. All statistical analyses were performed using SPSS (version 18.0, Chicago, IL, USA). Two sided p-values <0.05 were considered to be significant.

## Results

### Baseline characteristics and correlates with post-contrast T1

Mean age of reperfused AMI patients was 52.4±11.6 years. Eight patients had left anterior descending artery (LAD) territory and 10 had non-LAD territory myocardial infarction. Mean LV mass was 131.2±30.2 g and LV ejection fraction was 52.4±10.3%. Among 18 subjects, 17 were men. The cardiomyopathy patients consisted of four hypertrophic cardiomyopathy and nine dilated cardiomyopathy patients. Baseline patients’ characteristics are described in Table 1. In three AMI patients, only the upper four slices were analyzed due to poor delineation of non-infarcted myocardium or misregistration in two apical slices. The average T1 value of infarcted myocardium was significantly lower than that of non-infarcted myocardium (425.8±66.6 ms vs. 530.9±90.0 ms, respectively; p<0.001). Post-contrast T1 values from non-infarcted mid-ventricular LV slices (slice 3) inversely correlated with age in healthy controls (r=-0.833, p=0.010) and AMI patients (r=-0.424, p=0.079). Post-contrast T1 value of 3^rd^ slice (mid-ventricle) in cardiomyopathy was shorter than that of controls (511.0±91.5 ms vs. 612.6±82.4 ms, p<0.05).

**Table 1:** Baseline characteristics of study subjects

**Variables**	**AMI (n=18)**	**Cardiomyopathy (n=13)**	**Control (n=8)**
**Age,** yrs	52.4±11.6	48.2±13.8	35.1±10.3
**Men/women**	17/1	10/3	8/0
**Systolic blood pressure,** mmHg	110.0±17.0	115.3±11.7	
**Diastolic blood pressure,** mmHg	72.8±10.6	74.2±7.1	
**Heart rate, **bpm	78.0±13.7	77.7±15.4	
**Diabetes (%)**	4 (22)		
**LAD/RCA/LCx**	8/8/2		
**LV end-diastolic volume**, mL	141.9±31.3	182.7±71.3	
**LV end-systolic volume**, mL	68.7±26.1	125.2±102.2	
**LV mass**, g	131.2±30.2	155.1±53.5	
**LV ejection fraction**, %	52.4±10.3	44.9±28.7	

*LAD*, left anterior descending coronary artery; *RCA*, right coronary artery; *LCx*, left circumflex artery; *LV*, left ventricular.

### Time dependency of postcontrast-T1 value by slicing

Mean post-MOLLI scan time from apical slice to basal slice was 369.3±55.5 ms in AMI patients, 362.3±62.6 ms in cardiomyopathy patients and 339.5±38.1 ms in healthy controls (p=0.540). The T1 values of non-infarcted myocardium increased significantly from apex to base (from 523.1±99.5 ms to 561.1±81.1 ms, p=0.001), accompanied by increased LV cavity blood T1 (from 442.1±120.7 ms to 456.8±97.5 ms, p<0.001) over time (Figure 3). This phenomenon was applied to both LAD territory (from 545.1±74.5 ms to 575.7±84.0 ms, p<0.001) and non-LAD territory cases (from 501.2±124.5 ms to 549.5±81.3 ms, p<0.001) (Figure 4). It was also similarly applied to healthy volunteers (from 543.6±99.5 ms to 629.5±70.4 ms, p<0.001) and primary cardiomyopathy patients (from 455.4±125.6 ms to 514.7±86.2 ms, p=0.020) but not in infarcted myocardium (p>0.05). When we adjusted non-infarcted T1 values using LV cavity blood T1 values, the change of the corrected T1 was not significant among the slices from apex to base in AMI (from 1.17±0.18 to 1.25±0.13, p>0.05), cardiomyopathies (1.26±0.18 to 1.39±0.28, p>0.05) and healthy controls (1.17±0.09 to 1.17±0.14, p>0.05) (Table 2). Ve did not significantly change from apical slice to basal slice in the cardiomyopathy group (Table 3). In the four repeated scanning in AMI patients, T1 value of non-infarcted myocardium was not significantly different (slice 1, p=0.462; slice 2, p=0.134; slice 3, p=0.798; slice 4, p=0.600; slice 2, p=0.533; slice 6, p=0.093). In healthy controls, there was no significant systematic variability in post-contrast T1 value among myocardial segments within a slice (Table 4).

**Figure 3:**
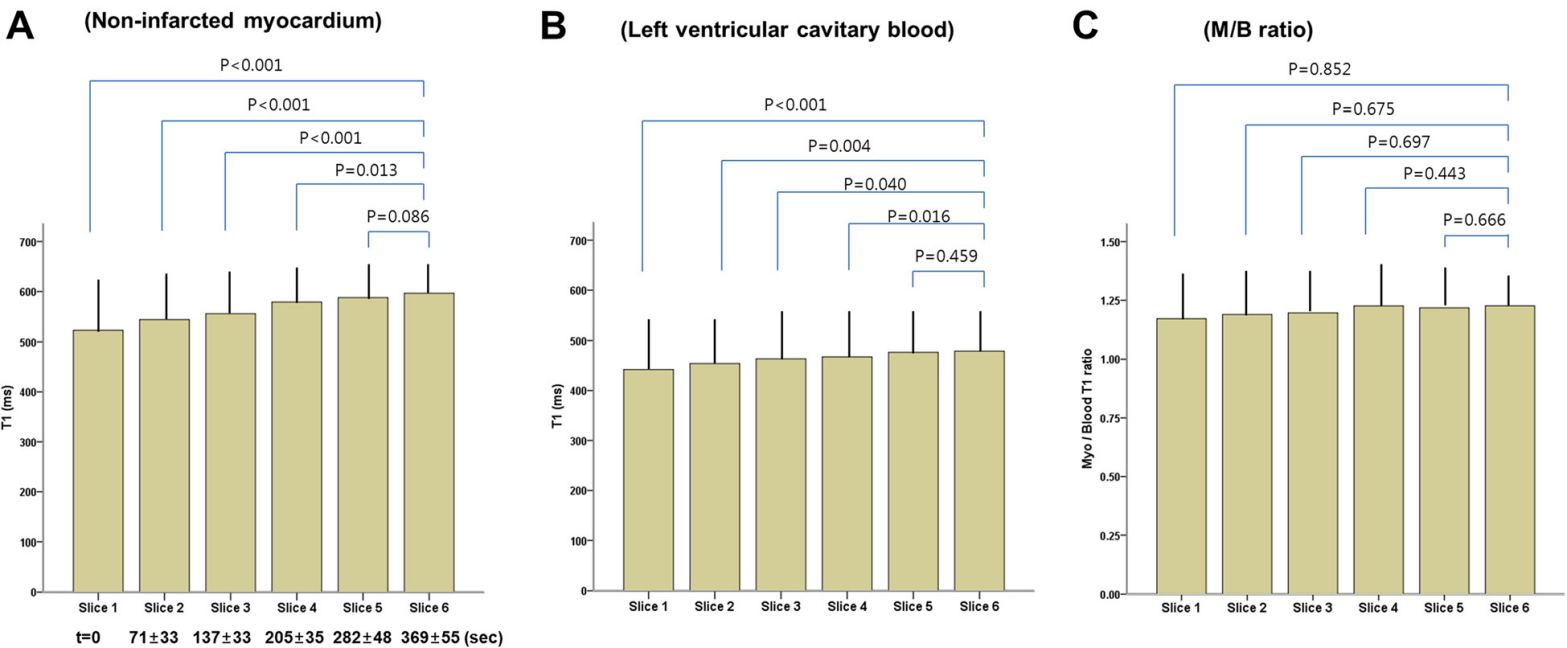
**Change of post-contrast T1 values from non-infarcted myocardium (A), left ventricular cavitary blood (B) and non-infarcted myocardium/blood (M/B) ratios (C) during slicing from apex to base in patients with acute myocardial infarction.** (t=mean time after acquisition of slice 1).

**Figure 4:**
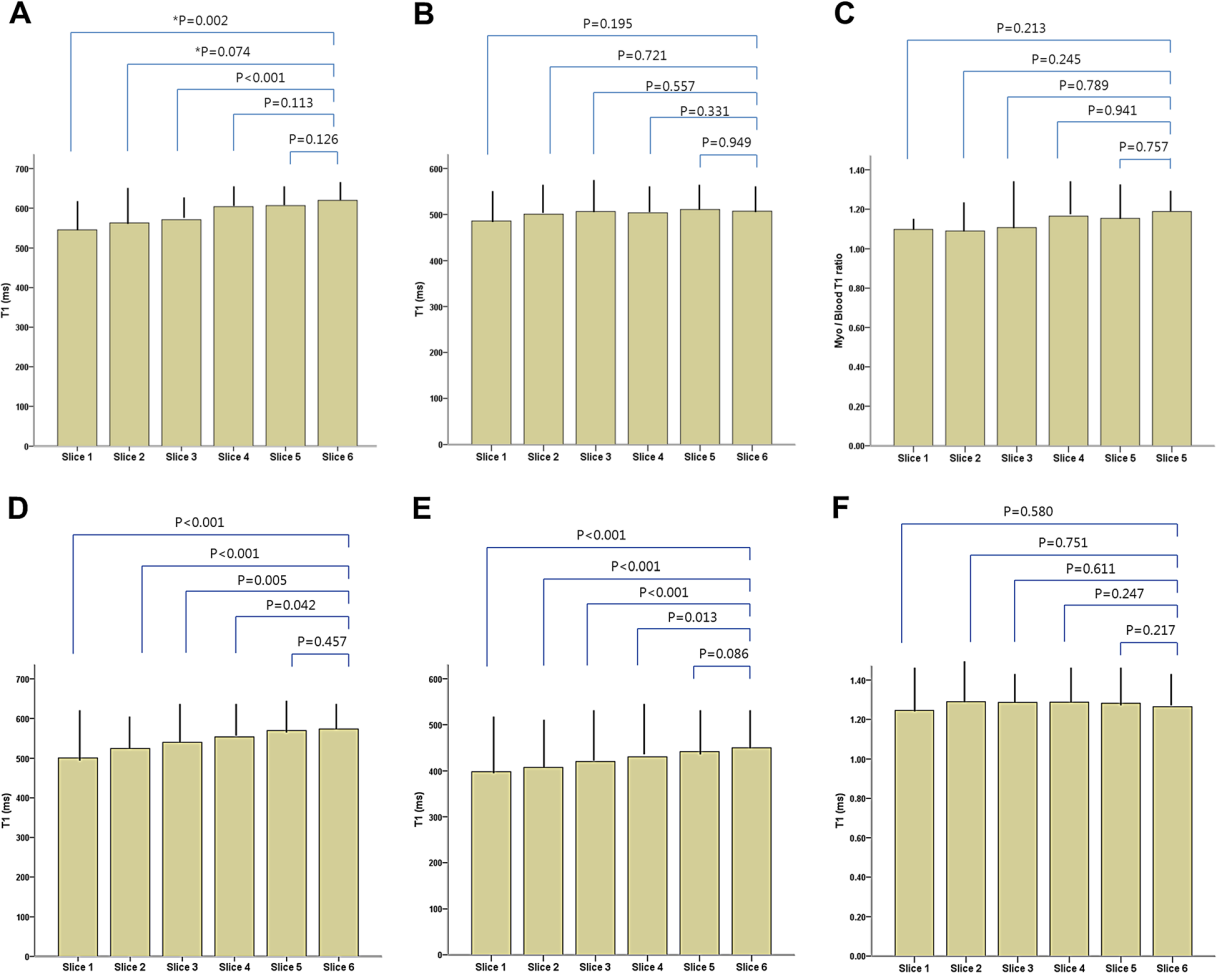
**Change of post-contrast T1 values from whole-, non-infarcted myocardium, blood and non-infarcted myocardium/blood ratios during slicing from apex to base in patients with LAD territory (A-C) and non-LAD territory (D-F) acute myocardial infarction.** LAD, left anterior descending artery, *Three cases were not analyzed due to poor delineation or misregistration in two apical slices.

**Table 2:** Fifteen-minute post-contrast T1 values (milliseconds) in healthy control individuals

	**Control (n=8)**					
	**Pre-contrast**			**Post-contrast**		
	**Myocardium**	**Blood**	*Time (sec)	**Myocardium**	**Blood**	**M/B Ratio**
**Slice 1**	987.5±17.4	1413.6±60.2	0	629.5±70.4	543.0±95.7	1.17±0.09
**Slice 2**	974.2±36.6	1410.4±65.4	62.7±18.8	627.4±77.4	542.9±103.7	1.17±0.10
**Slice 3**	973.0±19.1	1420.8±66.7	137.2±25.3	612.6±82.4	531.3±100.7	1.17±0.09
**Slice 4**	976.7±19.3	1427.1±60.8	195.5±21.6	598.6±92.7	517.1±112.1	1.18±0.10
**Slice 5**	960.0±16.0	1434.3±49.7	263.8±27.9	568.3±94.0	489.4±115.9	1.18±0.11
**Slice 6**	965.6±22.9	1414.6±51.3	339.5±38.1	543.6±99.5	475.7±123.5	1.17±0.14
**†p-value**	p>0.05	p>0.05		<0.001	0.033	>0.05

*Mean time after first slice acquisition of post-contrast T1 map; †denotes results from repeated measure ANOVA.

*M/B*, myocardium/blood T1 ratio.

**Table 3:** Pre-contrast and 15-minute post-contrast T1 values and Ve in cardiomyopathy patients

	**Cardiomyopathy (n=13)**				
	**Pre-T1 Myocardium (ms)**	**Post-T1 Myocardium (ms)**	**Blood (ms)**	**Ve (%)**	**M/B Ratio**
**Slice 1**	1127.4±282.7	514.7±86.2	421.8±99.1	34.7±17.1	1.26±0.18
**Slice 2**	1125.2±272.0	520.3±81.5	413.9±96.5	31.3±13.5	1.29±0.17
**Slice 3**	1113.1±272.5	511.0±91.5	407.1±104.4	31.0±12.1	1.30±0.14
**Slice 4**	1117.2±284.6	497.5±102.5	399.6±110.0	32.2±12.5	1.30±0.19
**Slice 5**	1163.5±265.6	473.5±115.4	366.2±150.9	34.0±12.2	1.36±0.29
**Slice 6**	1135.1±267.1	455.4±125.6	316.8±199.3	31.9±11.7	1.39±0.28
*p-value	>0.05	0.02	0.034	>0.05	>0.05

*denotes results from repeated measure ANOVA; M/B, myocardium/blood T1 ratio.

**Table 4:** Sixteen segmental value of 15- minutes post-contrast T1(ms)

	**Anteroseptal**	**Inferoseptal**	**Inferior**	**Inferolateral**	**Anterolateral**	**Anterior**	**P**
**Base** (slice #5 and #6)	641.4±91.0	643.44±79.6	626.8±58.7	629.1±55.6	638.5±65.9	656.3±75.4	>0.05
**Mid** (slice #4 and #3)	623.9±74.8	628.9±74.9	604.4±82.4	626.8±73.6	616.0±69.4	627.4±70.8	>0.05
**Apex**(slice #1 and #2)	*602.5±82.3		585.5±91.9	†597.4±75.1		595.5±71.3	>0.05

*Apical septum; †Apical lateral wall.

## Discussion

In this study we found that 15-minutes-post-contrast myocardial T1 values changed significantly, even during the brief time period of short axis-slicing in patients with AMI, or cardiomyopathy, and in healthy controls. However, the myocardial T1 to blood T1 ratio did not significantly change during the slicing as blood T1 values also changed in parallel to myocardial T1 signals. Calculated Ve from pre- and post-contrast MOLLI also did not significantly change from apex to base. Therefore, application of 15-minutes post-contrast T1 value, which is the currently-accepted standard protocol, to various cardiac diseases should be done with care and might benefit from blood T1 correction or calculated Ve.

Previous studies showed post-contrast T1 values can provide information regarding the degree of interstitial fibrosis that is not detected by current LGE techniques. [2,3,12] That research also provided useful information regarding the pathologic causes of diastolic dysfunction related to aging, hypertension, diabetes, or ischemic heart disease. [3] Our results also support this concept as non-enhanced post-T1 values were significantly and inversely correlated with age in healthy control individuals and AMI patients. Therefore, T1 values have great potential value in guiding treatment for diastolic heart failure, where large multi-center studies failed to achieve survival benefit from most medications [13]. Myocardial T1 value can be used as a surrogate imaging marker for monitoring the response to specific treatment such as anti-fibrotic agents or renin-angiotensin-aldosterone system blocking agents. However, to discriminate subtle change among study subjects or among different heart regions within a patient standardized methods that are less-affected by study environments are needed. Patients with AMI have various myocardial tissue characteristics such as replacement fibrosis, reactive interstitial fibrosis, peri-infarct tissue edema and remote healthy myocardium. To obtain regional tissue characteristics by T1 mapping, we need multiple short-axis LV slicing at the same position to LGE imaging. Although previous studies conducted to see changes in T1 before and at 5-, 10- and 45 minutes post-contrast injection [10], no previous studies have evaluated potential T1 changes during the brief period of slicing from apex to base, which is the same position as cine and LGE imaging.

In this study, we found non-infarcted myocardial T1 values changed significantly even in the short time period of slicing from apex to base in AMI patients. There might be arguments about the possible cause of T1 change in non-infarcted myocardium throughout the slicing, such as the influence of peri-infarct areas, regional myocardial wall motion abnormalities or related slow LV cavity flow. To assess these possibilities, we evaluated healthy control individuals and cardiomyopathy patients using the same protocol. We found similar results in all groups. Additionally, we separately analyzed the T1 value change in subgroups with LAD territory infarct and non-LAD territory infarct. We observed the same patterns of T1 change during the short time period of slicing from apexin to base in both subgroups. Actually mean duration of 6 slicing from apex to base took about 5-6 minutes regardless of disease subgroups and it was not so short. This is a significant problem because we want to detect subtle myocardial tissue changes using T1 relaxation time not only between patients but also among different inter-myocardial regions within an individual. Because we cannot obtain whole myocardial images with single breath-hold, three-dimensional imaging with current MOLLI sequence, some corrections should be considered. Currently, Ve eseems to be the most stable index for use in clinical studies. This idea is also supported by our study results. However, when considering the complex equations, several factors are known to influence pre-contrast T1 (i.e. heart rate, poor delineation of border), and small but significant Ve time dependency must be considered [10]. Post-contrast myocardial T1 after correction for blood T1 and equalizing external conditions is a convenient and practical approach to optimizing cardiac imaging.

### Study limitations

First, there was still variability of 15 minute post-contrast T1 values of non-infarcted remote myocardium among patients. This variability could reflect real differences of myocardial tissue character between subjects, or arise from subtle scanning time differences after contrast injection. Although we tried to obtain images exactly at 15 minutes after contrast injection in all study subjects, the influence of slightly different timing after contrast administration was not completely excluded. However, when considering post-contrast T1 of cardiomyopathy was shorter than that of healthy volunteers and repeated scanning did not show significant differences in non-infarcted myocardial post-contrast T1 values obtained with same time protocol, this variability mainly comes from tissue character. Secondly, although we used a motion-correcting algorithm for T1 mapping using 11 MOLLI images, artifacts when drawing ROIs could affect T1 values in cases with imprecise delineation of epicardial, endocardial or infarcted borders.

## Conclusion

In this study, we found that post-contrast 15-minute myocardial T1 value increased over time but the ratio of myocardial T1/blood T1 was rather stable. The Ve calculated using pre-, post- MOLLI sequence and blood hematocrit level, was stable throughout the entire myocardial region. Therefore, we recommend to use15-minute-post-contrast myocardial T1 values after correcting for blood T1 or calculated Ve when multiple slicing is conducted over time.

## Abbreviations

MOLLI: Modified Look-Locker Inversion Recovery; AMI: Acute Myocardial Infarction; Ve: Extracellular Volume Fraction; LV: Left Ventricular; LAD: Left Anterior Descending Coronary Artery; CMR: Cardiovascular Magnetic Resonance; ROI: Region Of Interest; FISP: Fast Imaging With Steady-State Precession; TR: Repetition Time; TE: Echo Time; LGE: Late Gadolinium Enhancement; FGRE: Fast Gradient Echo.

## Competing interest

The authors declare that they have no competing interest.

## Authors’ contribution

CEY, HSH and KTH: Designed study. Collected, analyzed and interpreted data. Statistical analysis. Wrote article. PCH: Collected, analyzed and interpreted CMR data. KHM, RSJ, HBK, YYW, MPK, KJY: Collected clinical and CMR data. YJH, CH: Study Coordination, CMR data collection. PMY, AG; Provided critical technical support and revised manuscript. All authors read and approved the final manuscript.
